# Klaus Michael Meyer-Abich: Was es bedeutet, gesund zu sein. Philosophie der Medizin: Carl Hanser Verlag, München, 2010, 640 Seiten, ISBN 978-3-446-23413-0

**DOI:** 10.1007/s10202-011-0096-8

**Published:** 2011-11-10

**Authors:** Dorothee Dörr

**Affiliations:** Europäische Akademie GmbH, Bad Neuenahr-Ahrweiler, Germany

There is no doubt that occidental conceptions of health and disease harbour Cartesian assumptions thus unfolding far-reaching social and political implications. In accordance with the opinion of medical historians, ethicists and epistemologists, the dualistic image of humanity (*Menschenbild*) exerted a determining impact on medical science and practice most notably since the dissemination of Descartes’ philosophy. Hallmark of this insight is the partition of body and mind that is grounded on the distinction between two fundamental substances: *res extensa* and *res cogitans*. The former stands for moved material of the outside-world, the latter for the immaterial inner-world, thus reducing the body-world to a pure extensional dimension as deduced from a conception aligned on physical principles. In his mechanistic physiology, Descartes compares man with a machine animated with spirit. Accordingly, the immaterial *res cogitans* corresponds to human consciousness, encompassing man’s capability of reasoning. This radical differentiation between two substances, identified to be constitutional for human beings, leads towards the unsolved question about the nexus between such exceptionally divergent materials—namely the claim of a conclusively substantiated explanation of the body and soul interaction. Descartes in turn assumes that the physical connection between these different structures is represented through the *glandula pinealis*—which he asserts to be the principal site of the soul—allowing the interaction of the brain (mind) with the body. Subsequently, the inherent systematic argumentative inconsistency of this theory stipulates the advancement of alternative explanatory models, that successively have been evolved by representatives of different Cartesian currents (e. g. occasionalists, interactionists).

The implementation of the dualistic principle in contemporary medical science becomes particularly evident in the fields of molecular genetics or neuroscience. A characteristic feature of modern scientific approach is the reconstruction of cause and effect relation—compliant with the mechanistic scheme of *actio* and *reactio*—as well as the assessment of regularities based on this nexus. Correspondingly, the question of the causality between mental events and physical-chemical brain processes is a key issue in neurobiological debates. From the anthropological view point, the reduction of man to his scientifically examinable brain functions consequently implies a deterministic definition of man—as sentenced by some exponents of cognitive sciences. Consistent with this comprehension, the human body corresponds to a *system of movements* that is accessible to an objective functional analysis. On the one side, this mechanistic reading of the human body allows a heuristic, methodologically compelling scientific analysis of the impact of medical technology on the human body, and on the other side, it excludes an ethical reflection on a responsible handling of these interventions because of the implicit lack of criteria from the anthropological insight (Gethmann-Siefert).

In line with these observations, the work of Meyer-Abich prominences and critically reflects the manifestations of the Cartesian metaphysical dualistic anthropology in modern medicine. The author contributes in a remarkable way to the research of history and effective history (according to Gadamer: *Wirkungsgeschichte*) of medicine, outlining a critical portrait of development and current practice of medicine and medical science. However, the title *Philosophy of Medicine* holds a promise of an encompassing, rational founded approach to medical practice, that the book does not redeem. Instead, the volume presents a naturalistic perspective of medicine which emphasizes but the psychosomatic viewpoint. Among the numerous illustrations of doctrines that influenced medical science, the content includes instructive lessons about the notion of Groddeck—renowned as founder of psychosomatic medicine—which points out the parallelism of physical and mental states experienced by patients in every course of any disease. Furthermore, to a great extent, Meyer-Abich follows the anthropological viewpoint (more precisely: psychosomatic groundwork) of Viktor von Weizsäcker emphasizing the social dimension of health and disease.

According to the author, the selfishness of today’s lifestyle in western welfare countries, which is strongly oriented towards the principle of individual autonomy, is not compliant with human nature, which instead requires a dedicated integration of mankind in his surrounding world. One basic need of human beings consists in the accomplishment of a balanced equilibrium between the individual selfhood (*Selbstsein*)—distinctly marked by the individual’s own world—and perceiving itself as an integrating component of the universal world (*togetherness* = *Mitsein*; notion originated by Heidegger and adopted by O. Schwemmer). Furthermore, Meyer-Abich argues that almost any disease is man-self-made, that is: today’s diseases are induced by individual misbehaviour and a disease propagating environment because of the disequilibrium between man and his surrounding nature. This approach holds convincing elements especially in regard to the knowledge about the multifactorial occurrence of leading diseases in occidental cultural circles. Notably, diseases of the cardiovascular system, diabetes type II, certain defined types of cancer, chronic back pain or the considerable increase in depressive disorders are triggered by individual predisposition and environmental influences. Although many of the underlying causal relations of diseases have been perceived long since, however, the claim of generalization of this conjunction to all diseases appears to be elusive. The book accentuates the importance of explanation to patients about the meaningfulness of ailments (*Sinnhorizont von Krankheit)*. In some parts, the author propagates even a strong medical paternalism: The aim is not merely to educate the individual to preserve his/her health condition, but moreover to unveil presumable disease propagating specific behaviours and to insist on counter measure lifestyle-attitudes for the individual. Moreover, the quest for the possibility of virtually eliminating the burden of disease for mankind by adopting over sound provisions occurs as highly controversial and scarcely accessible to reason. Although clearly opposed to the author’s psychosomatic approach, parallels to futuristic visions shared by scientists who believe imperturbably in scientific progress applicable for the eradication of human suffering come to mind, whereas the replicability of the insight that today’s medicine—as applied science—is primarily disease focussed (*Krankheitsmedizin)* and, therefore, it is not taking into view the preservation and *care of health* as primarily orientation but rather the reconstitution of health, meets current issues for the development of a sound-based medical care system. However, the consideration of medical prophylaxis as basic objective of the health care system strikes as a very ambitious goal, as it implicitly demands the promotion and prescription for the individual of *the* proper conduct of life.

The book’s claims ground on the psychosomatic current which allegeable by now influences today’s medical practice progressively and meaningfully and is renowned to be an essential part of the conclusive concept of modern integrative medicine. Hence, to some extent, Meyer-Abich’s perception strikes as outdated, dismissing the latest efforts in the fast evolving fields of modern medicine. In addition, it should be emphasized that overcoming of the dualistic concept is neither accomplished in conventional western mainstream medicine nor in the *psychosomatic* notion, as notably already implemented in its terminology, and extended through the endeavours of promoting a “*body*-*and*-*mind medicine*”.

In the aggregate, the book holds a considerable exposition of philosophical trains of thought and historically forwarded remarkable case reports, complemented by personal experience and intuitions of the author.

